# Reutilization of Reclaimed Asphalt Binder via Co-Pyrolysis with Rice Husk: Thermal Degradation Behaviors and Kinetic Analysis

**DOI:** 10.3390/ma16227160

**Published:** 2023-11-14

**Authors:** Hui Zhao, Bao Mi, Na Li, Teng Wang, Yongjie Xue

**Affiliations:** 1 State Key Laboratory of Silicate Materials for Architectures, Wuhan University of Technology, Wuhan 430070, China; 2 School of Environmental Engineering, Wuhan Textile University, Wuhan 430073, China; 2018001@wtu.edu.cn; 3 Engineering Research Centre for Clean Production of Textile Dyeing and Printing, Ministry of Education, Wuhan Textile University, Wuhan 430073, China

**Keywords:** reclaimed asphalt binder, co-pyrolysis, TG-FTIR analysis, iso-conversional method, combined kinetic analysis

## Abstract

Realizing the utilization of reclaimed asphalt binder (RAB) and rice husk (RH) to reduce environmental pollution and expand the reutilization technique of reclaimed asphalt pavement (RAP), co-pyrolysis of RAB with RH has great potential. In this study, the co-pyrolysis behaviors, gaseous products, and kinetics were evaluated using thermogravimetric analysis and Fourier transform infrared spectroscopy (TG-FTIR). The results showed that incorporating RH into RAB improved its pyrolysis characteristics. The interactions between RAB and RH showed initial inhibition followed by subsequent promotion. The primary gaseous products formed during co-pyrolysis were aliphatic hydrocarbons, water, and carbon dioxide, along with smaller amounts of aldehydes and alcohols originating from RH pyrolysis. All average activation energy values for the blends, determined through iso-conversional methods, decreased with RH addition. The combined kinetic analysis revealed two distinct mechanisms: (1) at the lower conversion range, the pyrolysis of the blend followed a random nucleation and three-dimensional growth mechanism, while (2) at the higher conversion range, the control mechanism transitioned into three-dimensional diffusion.

## 1. Introduction

As asphalt pavements near the end of their service life, a significant volume of reclaimed asphalt pavement (RAP) is generated globally due to the extensive maintenance and rehabilitation of deteriorated road surfaces [1]. The disposal of this substantial RAP waste has garnered significant public attention, driven by increasingly stringent environmental protection and sustainable development policies [2,3]. RAP typically contains approximately 4–6% binder and high-quality aggregate [4], and the reclaimed asphalt binder (RAB) is derived from bitumen recycled from RAP materials [5]. Ideally, RAPs can replace new asphalt binders and aggregates in specific proportions to create new asphalt mixtures. However, the aged RAB within RAP becomes notably harder, more brittle, and prone to cracking, posing a significant challenge that hampers the high-value recycling of RAP [6]. Applying a high content of RAP (over 30%) is approached with caution, as it adversely affects the water damage resistance, fatigue resistance, and cracking resistance of reclaimed asphalt pavements [7,8,9]. Furthermore, issues such as considerable gradation variability, poor adhesion to new asphalt, and uneven mixture blending have impeded the widespread adoption of high RAP content mixtures [10,11]. Despite concerted efforts to address these challenges [2,12,13], many highway agencies continue to opt for low RAP content or conventional mixtures to ensure more stable construction quality and mitigate potential shortcomings [14,15]. 

Despite the abundance of this valuable resource, some portions of reclaimed asphalt pavement (RAP) are still being disposed of in landfills, while another portion is being utilized in non-asphalt applications, such as embankments, subbases, bases, and shoulders [16]. Unfortunately, the majority of RAP is not being recycled to its full potential, and the failure to utilize it not only represents material waste but also poses an environmental hazard. The mineral aggregate within RAP can be reused as a new material, and the aged RAB can be centrally processed. In other words, to maximize the utilization of RAP, there is a pressing need to explore innovative techniques for RAB utilization. On the other hand, asphalt binder generated from the crude oil refining process consists of complex mixtures of macromolecular hydrocarbons and oxygenated organic compounds, hinting at its potential for reutilization in the recovery of energy. Pyrolysis is considered a viable thermal conversion technique for solid waste, with the capacity to generate high-added-value products such as gases, liquids, and solid energy [17]. Researchers have undertaken studies on the pyrolysis of fresh asphalt binder to better understand its thermal decomposition characteristics and apparent activation energy at high temperatures [18,19,20,21]. Few modifications of the asphalt binder structure are observed up to 300 °C, with thermal decomposition necessitating even higher temperatures, resulting in low energy efficiency drawbacks. Different from fresh asphalt binder, RAB contains a higher asphaltene content, rendering its thermal degradation more challenging. Therefore, it is imperative to find a renewable and environmentally friendly auxiliary fuel to improve the pyrolysis characteristics.

Recent reports highlight that the co-pyrolysis of biomass and coal presents a promising solution to address the challenges associated with handling biomass or coal separately [22,23,24,25]. Furthermore, the synergetic benefits of incorporating biomass into coal pyrolysis have been thoroughly investigated, positioning co-pyrolysis as a means of bridging the gap between fossil fuels and renewable fuels in terms of energy output [26]. Moreover, it is also shown that co-pyrolyzing biomass with various organic solid residues, such as waste tires [27], low-density polyethylene [28], and other industrial plastics [29], has the potential to enhance both the yield and quality of value-added products while reducing the risk of secondary contamination. Consequently, the quality of pyrolyzed products, gaseous phase emissions, and pyrolysis efficiency can be significantly improved by co-pyrolyzing biomass with carbon and hydrogen-enriched mixtures. Asphalt binder is a complex mixture consisting of distinct molecular groups of hydrocarbons and their derivatives [30], suggesting that co-pyrolysis of RAB with biomass holds promise for the conversion of energy sources. Furthermore, it has been demonstrated that co-pyrolysis processes can benefit from the presence of alkaline metals in biomass, which can serve as catalysts to expedite gasification [31]. Rice husk (RH), the most representative biomass, is produced approximately 134 million tons annually worldwide, but less than 10% of this volume is properly managed and disposed of [32]. Additionally, the abundant alkaline metals in RH are also considered to play a crucial role in the catalytic pyrolysis process [33]. However, to the best of our knowledge, so far, quite limited work has been performed on the pyrolysis of RAB and its blends with RH for the improvement of pyrolysis characteristics. The kinetic process and its co-pyrolysis mechanism remain largely unknown. 

The aim of this work is to explore the potential benefits of co-pyrolyzing RAB and RH. The TG-FTIR technology was employed to investigate the pyrolysis behaviors and determine the distribution of gaseous products from the pyrolysis of RAB and its blends. To gain insights into the reaction kinetics, the apparent activation energies were estimated through iso-conversional analysis of Flynn–Wall–Ozawa (FWO), Starink, and Friedman methods. The determination of the reaction mechanism is the application for the first time of the combined kinetic analysis to the study of RAB and its blends.

## 2. Materials and Methods

### 2.1. RAB and RH Materials

During the milling of the pavement of the FuYin highway in China, RAP materials were gathered from a stockpile of asphalt that had been reclaimed. The asphalt binder used in this construction site was SBS (styrene-butadiene block copolymers, 1301-1 linear structure) modified asphalt. ASTM D2172-05 [34] was used to extract RAB, which was subsequently recovered using ASTM D5404-03 [35]. RH obtained from a local grain processing plant was ground to powder with particles less than 200 μm in size. Table 1 shows the proximate, ultimate analysis, and main inorganic composition of samples of RAB and RH. RH has a high ash content (16.34%), while RAB has a high volatile matter content (87.62%). The larger volatile matter in RAB makes its potential better than RH for energy recovery.

### 2.2. Preparation of RAB and RH Blends

Using the mixer, the RAB sample was heated at 135 °C to a fluid condition, and then the addition of RH was mixed at 2500 rpm rotation speed for 30 min to ensure the RH was completely dispersed in the asphalt binders [36,37]. The blending of RAB and RH samples was formed by combining 10% and 30% (by weight) of RH, which were remarked as R9H1 and R7H3, respectively. The original RAB was tested as a blank sample. After mixing, mixtures were put into a metal cuboid mold with the size of 10 mm × 10 mm × 120 mm. After cooling the mold in a desiccator, the hardened blends of RAB and RH was removed from the mold and cut into cuboid with a size of 2 mm × 2 mm × 1.5 mm for further thermogravimetric analysis.

### 2.3. TG-FTIR Method and Instrument

The combined TG-FTIR analytical instrument is composed of thermogravimetry (Netzsch, STA449F3, Selb, Germany) and Fourier transform infrared spectrometry (Nicolet IS 50, Thermo Fisher Scientific, Waltham, MA, USA). At a heating rate of 10 °C/min, the samples tested with the mass of 10 ± 0.02 mg were heated from ambient temperature to 900 °C. The input gas was pure argon with a 100 mL/min gas flow rate. A capillary line from the TG analyzer sample point to the FTIR spectrometer gas cell was heated and maintained at 220 °C to keep gas products from condensing. With a spectrum region ranging from 650 cm^−1^ to 4500 cm^−1^, the FTIR resolution was 4 cm^−1^, and the spectrum scan frequency was 20 times per minute.

### 2.4. Pyrolysis Indices

The comprehensive pyrolysis index (CPI) was introduced to evaluate the pyrolysis characteristics and calculated as follows [38].

(1) $$CPI=\frac{D_{max}D_{mean}\left(1-M_{f}\right)}{T_{i}T_{max}\Delta T_{1/2}}$$

where $D_{max}$ is the maximum mass loss rate; $D_{mean}$ is the average mass loss rate; and $\Delta T_{1/2}$ is the temperature range corresponding to $D/D_{max}=0.5$ (half-peak width).

### 2.5. Interaction Analysis

The interactions of co-pyrolysis between the different fuels often play a decisive role in practical applications. The deviations between the theoretical TG and the test values of RAB-RH mixtures were selected to evaluate the interactions [39]. Assuming that there were no interactions during the co-pyrolysis of RAB and RH, theoretical values were calculated using Equation (3). Deviation values (%) were subsequently calculated using Equation (2) to evaluate the interaction degree.

(2) $$\Delta W=\frac{TG_{exp}-TG_{cal}}{TG_{cal}}\times 100\%$$

(3) $$TG_{cal}=x_{RAB}TG_{RAB}+x_{RH}TG_{RH}$$

Here, ΔW is the deviation, of which the positive and negative values correspond to the inhibitive and synergistic effects, respectively. $TG_{exp}$ is experimental mass loss of the blends. $TG_{cal}$ is obtained from the weighted calculation in accordance with the blending ratio of each individual feedstock in the blends at the same temperature.

### 2.6. Kinetic Theory

Kinetic analysis based on TGA (non-isothermal) data is a prevalent computational method. Pyrolysis is a heterogeneous process due to the thermal decomposition of tested samples to produce solid, liquid, and gaseous products. The heterogeneous reaction rate is provided by Equation (4)

(4) $$\frac{d\alpha }{dt}=k\cdot f\left(\alpha \right)$$

Here, $\frac{d\alpha }{dt}$ is a change in conversion rate over time. f(α) is the reaction model. α represents the conversion rate during the reaction process. k refers to the Arrhenius rate constant. α and k can be calculated using the following formula.

(5) $$\alpha =\frac{\left(m_{0}-m_{t}\right)}{\left(m_{0}-m_{f}\right)}$$

(6) $$k=Aexp\left(-\frac{E_{\alpha }}{RT}\right)$$

where $m_{0}$, $m_{f},$ and $m_{t}$ denote the initial mass, the final mass, and the transient mass of samples, respectively. A, $E_{\alpha }$, R, and T are the pre-exponential factor, the activation energy, the universal gas constant, and the sample absolute temperature, respectively. Under a constant heating rate β (β=dT/dt), Equation (4) can be transformed into the following form.

(7) $$\frac{d\alpha }{dT}=\frac{A}{\beta }exp\left(-\frac{E_{\alpha }}{RT}\right)f\left(\alpha \right)$$

Rearranging and integrating Equation (4) can obtain the equation as follows [40].

(8) $$G\left(\alpha \right)=\int_{0}^{\alpha }\frac{d\alpha }{f\left(\alpha \right)}=\frac{A}{\beta }\int_{0}^{T}exp\left(-\frac{E_{\alpha }}{RT}\right)dT=\frac{AE_{\alpha }}{\beta R}P\left(X\right)$$

where G(α) represents an integral form of the reaction model and $X=E_{\alpha }/RT$. However, P(X) does not have a precise solution as an integral form of temperature. It can be solved using the numerical approximation methods. 

#### 2.6.1. Iso-Conversional Analysis

In the iso-conversional method, two approaches, namely the differential method and the integral method, both estimate activation energy at an increasing conversion rate, which overcomes the requirement of determining the reaction models [41].

The approximation provided by Doyle was used in the FWO method for the estimation of temperature integral P(X) [42,43,44]. And then, the FWO equation can be stated as follows:

(9) $$\ln\beta =\ln\left(\frac{AE_{\alpha }}{RG\left(\alpha \right)}\right)-5.3305-1.052\left(\frac{E_{\alpha }}{RT_{\alpha }}\right)$$

According to Equation (9), at each degree of α, lnβ and $1/T_{\alpha }$ corresponding to three heating rates (10 °C/min, 20 °C/min and 30 °C/min) are fitted into a straight line. The activation energy $E_{\alpha }$ can be determined by calculating the slope, $-1.052E_{\alpha }/R$. The FWO equation is the most commonly used in the integral iso-conversional method. Starink developed another approximation of P(X) for determining activation energy more precisely [45,46]. The equation can be provided as follows:

(10) $$\ln\left(\frac{\beta }{T_{\alpha }{}^{1.92}}\right)=C_{S}-1.0008\left(\frac{E_{\alpha }}{RT_{\alpha }}\right)$$

Similar to FWO methods, $E_{\alpha }$ can be determined by plotting $\ln\left(\beta /T_{\alpha }{}^{1.92}\right)$ versus $1/T_{\alpha }$ at an equivalent α for each heating rate and then $E_{\alpha }$ can be calculated using the slope of $-1.0008E_{\alpha }/R$. 

By taking natural logarithms on both sides of Equation (7), the Friedman equation as one typical differential method is obtained and can be written as follows [47]:

(11) $$\ln\left(\frac{d\alpha }{dt}\right)=\ln\left(Af\left(\alpha \right)\right)-\frac{E_{\alpha }}{RT_{\alpha }}$$

$E_{\alpha }$ at a fixed value of α can be determined from the slope of $-E_{\alpha }∕R$ by plotting $\ln\left(\beta \frac{d\alpha }{dT}\right)$ against $1/T_{\alpha }$.

#### 2.6.2. Combined Kinetic Analysis

Rearranging Equation (7) with natural logarithms taken on both sides can provide the following form.

(12) $$\ln\left(\frac{d\alpha /dt}{f\left(\alpha \right)}\right)=\ln\;A-\frac{E_{\alpha }}{RT_{\alpha }}$$

The determination of activation energy is a fundamental aspect of solid-state reactions, as it provides valuable insights into the underlying reaction mechanisms and facilitates the optimization of reaction processes. To this end, the selection of an appropriate f(α) function is critical for producing a linear plot. Table S1 summarizes several typical f(α) functions found in the literature, along with their corresponding kinetic mechanisms. It is important to note that the selection of the proper f(α) function is subject to a significant limitation. Specifically, the f(α) functions proposed in the literature are often developed under idealized physical conditions and may not fully capture the complexity of every solid-state reaction. Consequently, deviations from the theoretical kinetic models are expected due to various factors. To mitigate these limitations, a procedure has been developed, which involves using a generalized expression for f(α) [48].

(13) $$f\left(\alpha \right)=c{\left(1-\alpha \right)}^{n}\alpha^{m}$$

This expression is a modified form descended from the famous Sestak–Berggren empirical equation [49]. It has been shown that by simply adjusting the c, m, and n parameters, it can match most of the typical reaction models, including its deviations from the ideal conditions [48,50].

Combining and rearranging Equations (12) and (13) gives the following basic equation for the combined kinetic analysis.

(14) $$\ln\left(\frac{d\alpha /dt}{{\left(1-\alpha \right)}^{n}\alpha^{m}}\right)=\ln\;cA-\frac{E_{\alpha }}{RT_{\alpha }}$$

Combined kinetic analysis (CKA) is a powerful technique for determining both the kinetic parameters and kinetic model of a reaction without the need for any prior assumptions regarding the reaction mechanism. CKA is able to accurately fit experimental data obtained from any linear heating rate, making it a versatile and valuable tool for investigating complex reaction systems. An optimization approach based on the Pearson linear correlation coefficient between the left-hand side of Equation (14) and $1/T_{\alpha }$ was employed as the objective function to determine the optimal values of m and n. The values of $E_{\alpha }$ and ln cA were then calculated from the slope and intercept of the best-fit line, respectively. It should be noted that while neither m nor n have any physical significance, the resulting f(α) function provides an accurate fitting equation. By comparing the calculated f(α) function with the theoretical reaction mechanism function, valuable insights can be gained into the type of kinetic mechanism driving the reaction process. CKA generates kinetic triplets that are nearly equivalent to those obtained by the iso-conversional method, which is widely used for exploring the thermal decomposition of polymers [51,52,53]. 

### 2.7. Experimental Procedure

**Figure 1:**
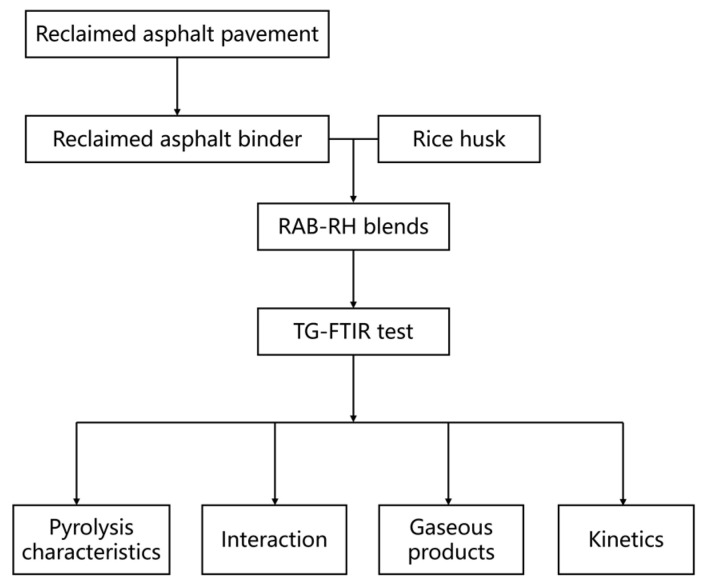
Flowchart of the experimental procedure.

Figure 1 illustrates the experimental procedure for this study. Initially, RAB is extracted and recovered from the milled RAP material. Subsequently, high-speed shearing is employed to evenly mix RAB and RH, resulting in mixtures with varying blending ratios. Finally, the pyrolysis characteristics, interactions, gaseous products, and pyrolysis kinetics of the RAB-RH mixtures are analyzed through the TG-FTIR test to demonstrate the potential of thermally utilizing the RAB-RH mixture.

## 3. Results and Discussion

### 3.1. TG-DTG Analysis

#### 3.1.1. Pyrolysis Characteristics

**Figure 2:**
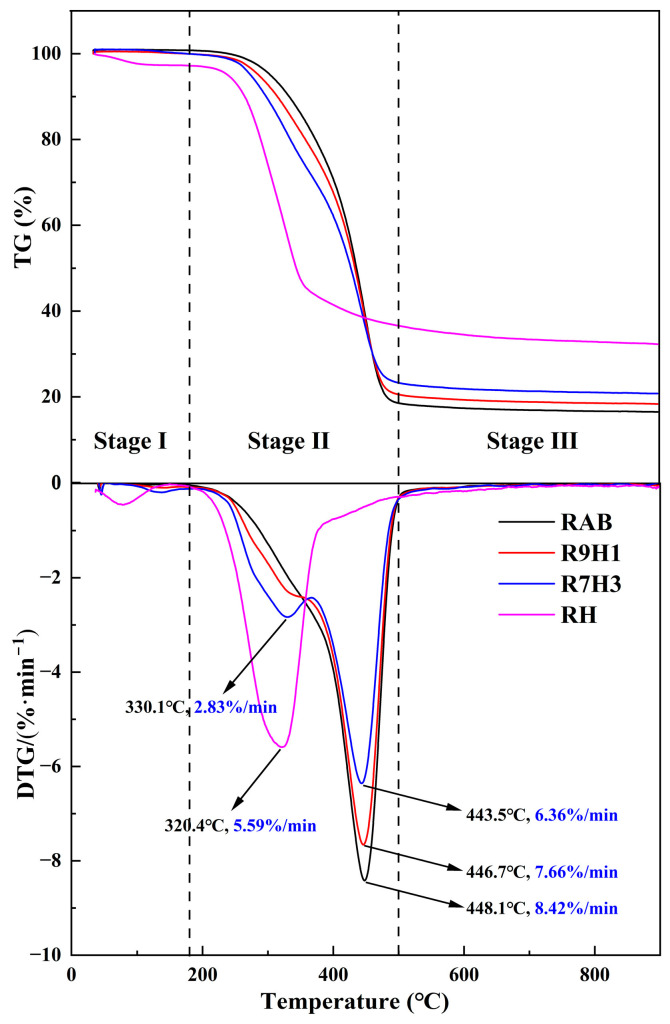
TG-DTG curves of samples pyrolyzed at a heating rate of 10 °C/min.

The TG and DTG curves of RH, RAB, and their blends are presented in Figure 2. The pyrolysis process of samples can be classified as the three processes of dehydration (<180 °C), pyrolysis (180–500 °C), and mineral decomposition (>500 °C) as the temperature increases. For RAB, a single DTG peak indicates that only the degradation and decomposition of organic matter occur. RAB decomposition predominantly takes place within the temperature range of 180–500 °C with a significant mass loss of 85.21%. In the initial stage (180–400 °C) of the RAB thermal decomposition reaction, weaker chemical bonds are destroyed due to the lower temperature, and the DTG curve decreases slowly. Subsequently, the RAB begins to decompose rapidly, caused by the breakage of strong chemical bonds with the increase in temperature, reaching a maximum mass loss rate of 8.42%/min at 448.1 °C. Meanwhile, two obvious peaks could be observed in the DTG curve of RH, corresponding to the moisture evaporation as well as the decomposition of hemicellulose, cellulose, and partial lignin [54,55]. The maximum mass loss rate of 5.59%/min is observed at 320.4 °C.

For the co-pyrolysis of blends, the curves display between RAB and RH curves with a similar trend of mass loss. It could be observed that a slight mass loss at temperatures below 180 °C due to the release of light volatiles and the evaporation of water in the feedstock. Furthermore, it is evident that the DTG peak temperature in this process shifts to high temperature when compared to RH, a phenomenon linked to an increase in diffusion resistance [56]. Within the temperature range of 180 °C to 500 °C, two distinct peaks emerge, indicative of differing pyrolysis characteristics between RH and RAB. The shoulder peak occurs in the temperature range of 180–400 °C, primarily associated with the decomposition of RH. A more significant peak between 400 and 500 °C results from the concurrent decomposition of both RH and RAB, with RAB dominating the process. Notably, as the proportion of RH incorporated into RAB increases, there is a corresponding increase in mass loss at the shoulder peak, coupled with a decrease in mass loss at the major peak. Detailed pyrolysis characteristic parameters for RH, RAB, and their blends are provided in Table 2.

As shown in Table 2, the initial decomposition temperatures of RH and RAB are measured as 271.9 °C and 376.3 °C, respectively. RAB exhibits an approximately 104 °C higher value than RH, indicating the devolatilization of RAB presents a greater challenge compared to RH. This disparity can be attributed to the fact that RAB is composed of macromolecular organic compounds with intricate structures. Consequently, the energy-intensive rupture of chemical bonds in RAB necessitates higher temperatures for decomposition. With the incorporation of RH increasing, the initial decomposition temperatures of blends decrease significantly compared with RAB, implying that the devolatilization of RH dominates the initial decomposition of the mixtures. Meanwhile, the terminated temperatures of blends become higher, indicating that the mineral decomposition of RH delays the reaction termination. It can also be found that the temperature at the maximum mass loss rate decreases slightly from 448.1 °C to 443.5 °C, probably suggesting that the degradation of RAB present in the blends with RH is advanced. Furthermore, the residue mass increases from 16.6% to 21.1%, primarily due to the high ash content of RH. The higher the CPI value, the more vigorously samples decompose. The addition of RH increases the CPI value from 5.61 to 8.03 × 10^−11^/min^2^ °C^3^, suggesting that the more content of RH in the blends, the higher the reactivity of the blends. Therefore, the co-pyrolysis of RAB and RH not only compensates for the limitations of RAB pyrolysis but also effectively enhances reactivity. 

#### 3.1.2. Interactions between RAB and RH

**Figure 3:**
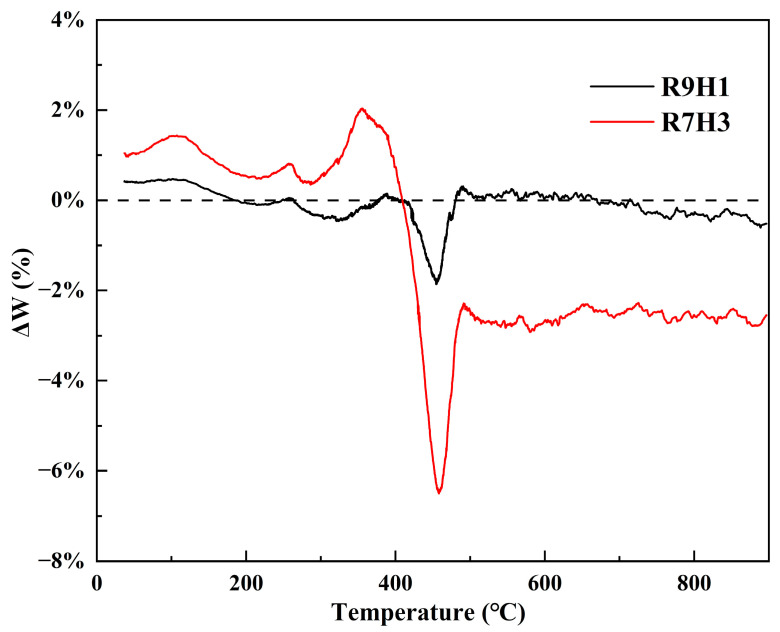
Deviation of experimental TG curves of blends with calculated curves using TGA data of individual samples (Equation (2)) at a heating rate of 10 °C/min.

The deviation varying with temperature between experimental and calculated TG curves is illustrated in Figure 3, and both blends present similar trends. With regard to R9H1, the deviation is mainly within ±0.5%, which has a low statistical significance. However, a stronger negative deviation can be found in the temperature range of 440 °C to 460 °C, which corresponds to the rapid decomposition of RAB components in the blends, indicating the incorporation of RH at a 10% dosage exhibits a certain synergistic effect for the thermal degradation of RAB. It should be noted that the R7H3 sample significantly displays the negative deviation and positive deviation. The positive deviation occurs from room temperature to about 400 °C, and the maximum deviation appears at about 340 °C, which is dominated by the decomposition of RH, suggesting that inhibitory effects between RAB and RH exist in this stage. This may be attributed to the fact that RAB is converted to flowing and viscous asphalt during the initial stages of thermal degradation, and these asphalts coat the RH powder, rendering the volatile matters from RH difficult to release at lower temperatures [57]. With the temperature increasing, the deviation begins to decrease to a negative value, indicating the synergistic effect between RAB and RH, and reaches the maximum negative value at about 455 °C. When the temperature exceeds 400 °C, the asphalt in RAB decomposes into gas and residue, reducing the diffusion resistance of the blends. Furthermore, the alkaline earth metals in RH ash have a catalytic effect on the thermal decomposition of hydrocarbons in RAB [58]. Meanwhile, the synergistic effect observed at above 500 °C may be attributed to the presence of inorganic non-metallic elements (calcium, magnesium, and potassium), which promote the carbonization process [59].

### 3.2. FTIR Results of Gaseous Products

**Figure 4:**
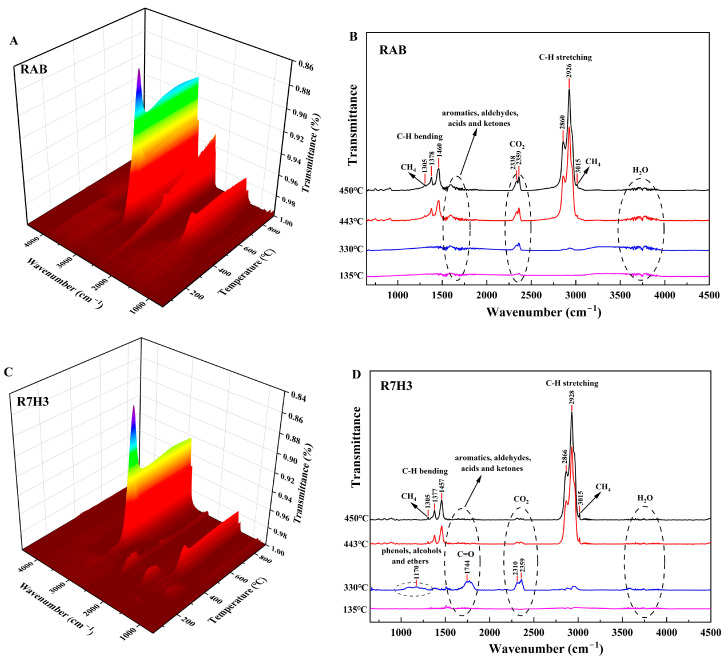
Three-dimensional FTIR spectra for volatiles of RAB (**A**) and R7H3 (**C**) and two-dimensional FTIR spectra at the selected temperature of RAB (**B**) and R7H3 (**D**).

The sample of R7H3, which possesses a significant synergistic effect, is selected to evaluate the release characteristics of gaseous products. The 3D FTIR diagram of RAB and R7H3 pyrolyzing at 10 °C/min is illustrated in Figure 4A,C, respectively. Results show that RAB and R7H3 exhibit similar absorption bands with the highest intensities observed at temperatures between 400 and 500 °C. This temperature range aligns with where the TG-DTG curves (Figure 2) exhibit their maximum mass loss rate. The peak temperature of FTIR is consistent with the peak temperature of DTG. Meanwhile, during this temperature range, the remarkable characteristic infrared absorption band with the highest absorbance at 3000–2750 cm^−1^ owing to the vibration of the C-H bond can be clearly observed, indicating the hydrocarbons are the main volatile products for RAB and R7H3 during pyrolysis. To further recognize the difference in volatiles, the 2D FTIR spectra for gaseous products of RAB and R7H3 at several specific peak temperatures, as determined by TG-DTG curves, and along with the typical bands with the possible compounds, are shown in Figure 4B,D, respectively.

The FTIR spectrum of RAB pyrolysis is shown in Figure 4B. Several noticeable absorption bands distributed to 3800–3500 cm^−1^, 3100–2750 cm^−1^, 2500–2250 cm^−1^, 1900–1500 cm^−1^, and 1500–1250 cm^−1^ can be observed. The bands at 3800–3500 cm^−1^ are related to the O-H bond stretching vibration, indicating the releases of H_2_O from the pyrolysis process [60], which is attributed to the evaporation of moisture in the samples, as well as the dehydration reactions of various oxygen-containing groups in the pyrolysis process with the temperature increasing [61]. The characteristic bands at 2500–2250 cm^−1^ indicate the formation of CO_2_. The release of CO_2_ may be ascribed to the thermally labile functional groups, such as carboxyl and carbonyl groups breaking and reforming [62]. Two stronger absorption bands at 3100–2750 cm^−1^ and 1500–1250 cm^−1^ are respectively associated with the stretching vibration and bending vibration of the aliphatic C-H bond, suggesting the release of light aliphatic gases, especially the formation of CH_4_, as indicated by characteristic bands at 3015 cm^−1^ and 1305 cm^−1^ [63,64]. The absorption band between 1750 cm^−1^ and 1500 cm^−1^ represents the stretching vibrations of aromatics, aldehydes, acids, and ketones [65]. Therefore, it can be inferred that the primary volatiles during RAB pyrolysis consist of substantial amounts of light aliphatic compounds, including methane, some inorganic small molecules such as H_2_O, CO_2_, and small amounts of aromatics, aldehydes, etc.

The gas evolution during co-pyrolysis of R7H3 can be divided into two distinct temperature regions. In the first temperature region (100–360 °C), corresponding to the pyrolysis of cellulose and hemicellulose in RH, several new absorption bands can be clearly observed in Figure 4D. The peak at 1774 cm^−1^ between 1850 and 1600 cm^−1^ absorption band is attributed to the C=O stretching vibrations induced by carboxylic acids, aldehydes, and ketones [66]. The weak absorption band at 1300–1000 cm^−1^ is associated with the stretching vibration of C-O functional groups present in phenols, alcohols, and ethers. In the second temperature region (360–500 °C), the gases produced are primarily ascribed to the decomposition of organic matter in RAB, releasing gaseous hydrocarbons as the predominant products.

### 3.3. Kinetic Analysis

#### 3.3.1. Estimation of Activation Energy

**Figure 5:**
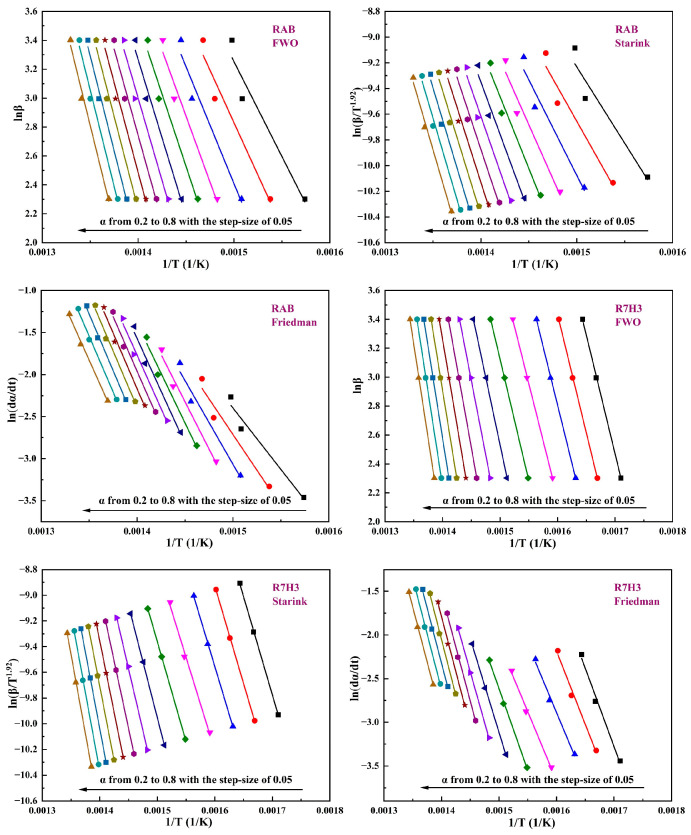
Linear fitting plots via three iso-conversional pathways for RAB and R7H3.

Figure 5 depicts the Arrhenius plots using FWO, Starink, and Friedman methods. Considering the thermal decomposition of asphalt might be unstable at the beginning and ending periods, the range of α employed here is from 0.2 to 0.8 with a step of 0.05 [67,68]. $E_{\alpha }$ is calculated by the slope of linear fitting curves. The fitting degree (R^2^) of R7H3 is more than 0.99, suggesting the reliability of the fitting results. Despite the fact that the linear fitting degree of RAB is not excellent (0.95 < R^2^ < 0.97) within the range of α from 0.2 to 0.3. The fitting results for RAB still provide valuable insights for estimating $E_{\alpha }$.

**Figure 6:**
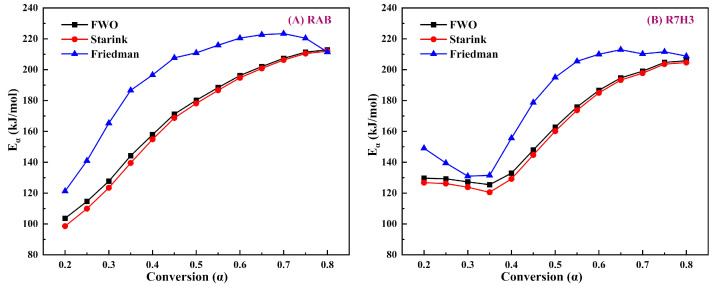
$E_{\alpha }$ versus α curves of (**A**) RAB and (**B**) R7H3 obtained using the iso-conversional methods.

$E_{\alpha }$ is the minimum amount of energy required to initiate the reaction. The distribution of the $E_{\alpha }$ corresponding to different degrees of conversion is shown in Figure 6. $E_{\alpha }$ values estimated for RAB and R7H3 using the FWO and Starink methods exhibit a similar trend and are almost overlapping. In contrast, $E_{\alpha }$ values obtained from the Friedman method differ significantly from those by the FWO and Starink methods. This difference may be attributed to the inherent features of the differential method used [69]. The $E_{\alpha }$ curves for RAB and R7H3 show clear variations with respect to α, indicating that the pyrolysis process of the sample is too complex to be characterized by a single-stage reaction [70]. For the pyrolysis of RAB, $E_{\alpha }$ values primarily represent an upward trend with the increase in α, because as the pyrolysis progresses, the organics in asphalt with high thermal stability demand more energy to break their chemical bonds [71]. The average values of $E_{\alpha }$ obtained from FWO, Starink, and Friedman methods are 170.61, 168.02, and 195.69 kJ/mol, respectively.

The initiation reaction $E_{\alpha }$ (0.1 <  α  < 0.3) of the R7H3 mixture is higher than that of RAB. This implies that the co-pyrolysis of blends at temperatures ranging from 280 °C to 340 °C demands more energy, primarily associated with the hemicellulose or cellulose decomposition from RH [58,72]. This could be caused by the fact that the organic matter with simple structures that decompose initially in RAB has less thermal resistance than that in RH. Moreover, RH powder is wrapped in viscous asphalt in RAB, further inhibiting the release of volatiles during the initial stages of RH pyrolysis. Nevertheless, the later stages of the co-pyrolysis process prove to be beneficial due to the decreasing value of $E_{\alpha }$, which is caused by the porous structure of biochar formed by pyrolysis of RH in the early process and catalysis of alkali metals in the ashes [66]. It is also indicative that pyrolysis reactions easily progress within the 340–465 °C temperature range, corresponding to the main decomposition of organic matter from RAB. The average $E_{\alpha }$ values of R7H3 obtained from FWO, Starink, and Friedman methods are 163.25, 160.74, and 179.98 kJ/mol, respectively, which are slightly lower than those for RAB. The inhibitory effects exist in the initial reactions, while the synergistic effects present in the later reaction stages are found to be consistent with the earlier discussion on the interaction between RAB and RH. In summary, the addition of RH enhances the value of the initial $E_{\alpha }$, but considering the later promotion effects on RAB pyrolysis, it can be concluded that mixing RH is beneficial to the overall pyrolysis of RAB.

#### 3.3.2. Determination of Reaction Mechanisms

**Figure 7:**
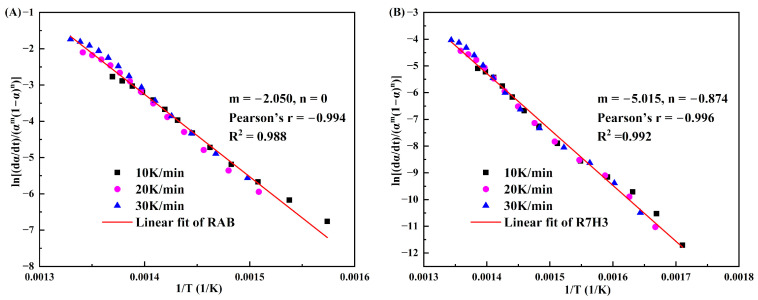
Combined kinetic optimization results of global reaction model for (**A**) RAB and (**B**) R7H3.

Figure 7 shows the fitting results of RAB and R7H3 pyrolysis using Equation (14) optimization via CKA. As can be seen, the linear regression Pearson’s coefficients of RAB and R7H3 are −0.994 and −0.996, respectively, indicating that the combined kinetic optimization results are in suitable agreement with the experimental data.

**Figure 8:**
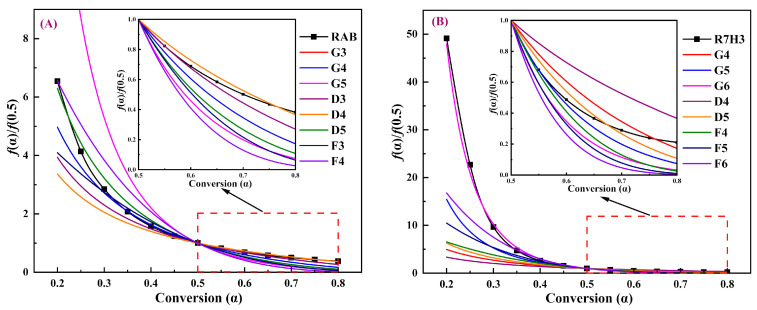
Comparisons of normalized curves at α = 0.5 of obtained kinetic models for (**A**) RAB and (**B**) R7H3 with the theoretical mechanism models.

The main kinetic parameters obtained from the iso-conversional method and CKA are detailed in Table 3. The $E_{\alpha }$ values calculated by the slope of the linear fits following the combined kinetic optimizations of RAB and R7H3 are 188.64 ± 3.45 kJ/mol and 174.03 ± 2.51 kJ/mol, which closely agrees with the average values obtained from the Friedman iso-conversional analysis. This alignment primarily stems from the fact that both calculation methods avoid systematic errors associated with integral approximation. The functions obtained from the combined kinetic analysis, i.e., $f\left(\alpha \right)=\alpha^{-2.05}$ for the pyrolysis of RAB and $f\left(\alpha \right)={\left(1-\alpha \right)}^{-0.874}\alpha^{-5.015}$ for the co-pyrolysis of R7H3 were compared with the commonly used conversion functions in Table S1 (such as nucleation and growth, chemical reaction, and diffusion), and the results are shown in Figure 8.

From Figure 8A, it can be found that when α = 0.2–0.3, the f(α)/f(0.5) curve for RAB closely approximates the D5 theoretical model (Zhuralev–Lesokin–Tempelman equation) with a three-dimensional diffusion mechanism. In the α range of 0.3–0.5, the experimental data of RAB matches well with the G4 theoretical mechanism, described by the Avrami–Erofeev equation (n = 2), suggesting a process of random nucleation and growth. However, at the higher α range of 0.5–0.8, the thermal decomposition mechanism for RAB pyrolysis is more inclined to the D4 (Brounstein–Ginstling equation) mechanism, which pertains to a three-dimensional diffusion process.

Moreover, it can be readily seen from Figure 8B that the thermal decomposition mechanism for the co-pyrolysis of R7H3 follows the G6 model in the α range of 0.2–0.5, corresponding to a random nucleation and nucleus growth process provided by the Avrami–Erofeev equation (n = 4). Inferred from the deviation between the experimental busbar of R7H3 and the theoretical mechanism, the D5 theoretical model with a three-dimensional diffusion mechanism is the most likely to describe the thermal decomposition process of R7H3 in the higher α range of 0.5–0.8. It is observed that with the introduction of RH, the value of the Avrami exponent (n) for the prophase thermal decomposition reaction of R7H3 increases from 2 to 4 while the later reaction of polycondensation and carbonization are both dominated by three-dimensional diffusion. The value of n is influenced by the nucleation rate, the geometry of the nuclei, and the growth mechanism (diffusion or chemical reaction), and its maximum value is 4 [73]. The G4 mechanism with n = 2 of RAB can be interpreted by a constant nucleation rate and the nuclei growth controlled by the diffusion of migrating substances, restricted to two dimensions where the nucleation sites are distributed randomly on the RAB surface. On the other hand, the G6 mechanism with n = 4 for R7H3 represents the three-dimensional nuclei growth controlled by a chemical reaction at the phase boundary with a constant nucleation rate [73,74]. This might be attributed to the fact that the addition of RH changed the original homogeneous nucleation system, and these heterogeneous RH served as preferential nucleation sites for unhindered nuclear growth controlled by the phase-boundary reaction.

## 4. Conclusions

Adding RH to RAB significantly improved the pyrolysis characteristics of the blends, as evidenced by the decrease in the $T_{i}$ and the increase in the CPI value. The primary pyrolysis products of RAB and R7H3 blends consisted of aliphatic hydrocarbons, water, and carbon dioxide, while ethers and alcohols derived from RH additive pyrolysis increased the species of gaseous products with small molecules in blends, providing evidence for the potential conversion of these materials into energy gas via pyrolysis. The analysis of interaction and activation energy analysis in the blends both revealed an initial reaction inhibition followed by promotion effects. The decline in activation energy for the blends further demonstrated that the addition of RH facilitated the overall pyrolysis reaction. The pyrolysis mechanism, as determined by the CKA kinetic model, could be elucidated as follows: the pyrolyzed reaction of R7H3 was initially dominated by random nucleation and three-dimensional nuclei growth when α = (0.2–0.5), resulting from the preferential nucleation sites provided by RH. Subsequently, when α reached (0.5–0.8), the pyrolyzed reaction was taken over by three-dimensional diffusion. The results of this study offer important insights for future research on the pyrolysis of RAB that is extracted and recovered from RAPs into value-added products. Additionally, this study also provides useful information for the large-scale adoption of pyrolysis technology to treat RAB.

## Figures and Tables

**Table 1:** The properties of RAB and RH (dry basis, wt.%).

Item	RAB	RH
Ultimate analysis		
Carbon	82.08	38.12
Hydrogen	9.15	5.05
Oxygen ^a^	1.13	43.14
Nitrogen	0.48	0.56
Sulfur	5.13	0.18
Proximate analysis		
Volatile	87.62	65.44
Fixed carbon ^a^	11.83	18.22
Ash	0.55	16.34
Ash analysis		
Na_2_O	-	0.25
K_2_O	-	3.86
CaO	0.28	2.38
MgO	0.02	0.75

^a^: Calculated by difference.

**Table 2:** Pyrolysis characteristic parameters at a heating rate of 10 °C/min.

Samples	$T_{i}$ (°C)	$T_{f}$ (°C)	$T_{max}$ (°C)	$M_{f}$ (%)	CPI (10^−11^/min^2^ °C^3^)
RAB	376.3	520.2	448.1	16.6	5.61
R9H1	273.9	531.1	446.7	18.5	6.74
R7H3	256.1	550.9	443.5	21.1	8.03
RH	271.9	669.3	320.4	32.8	4.48

$T_{i}$, the initial decomposition temperature determined by the intersection of the TG curve tangent and the horizontal curve; $T_{f}$, the terminated temperature where the mass loss of decomposable matters in the samples reached 98%; $T_{max}$, the peak temperature according to the maximum mass loss rate; $M_{f}$, the residue mass at 800 °C; CPI, comprehensive pyrolysis index.

**Table 3:** Main kinetic parameters for the thermal decomposition reaction of RAB and R7H3.

Samples	Combined Kinetic Analysis				Friedman Method
	$E_{\alpha }$ (kJ/moL)	ln cA (s^−1^)	m	n	Average $E_{\alpha }$ (kJ/moL)
RAB	188.64 ± 3.45	28.51 ± 0.59	−2.050	0	195.69
R7H3	174.03 ± 2.51	24.02 ± 0.45	−5.015	−0.874	179.98

## Data Availability

Data are contained within the article.

## Supplementary Materials

The following supporting information can be downloaded at: https://www.mdpi.com/article/10.3390/ma16227160/s1, Microsoft Word v. 2016/2021 Table S1: Algebraic expressions of functions f(α) proposed in the literature and their corresponding mechanisms [75,76,77,78].
