# Supplementary material for: Reutilization of Reclaimed Asphalt Binder via Co-Pyrolysis with Rice Husk: Thermal Degradation Behaviors and Kinetic Analysis
Source: Materials (Basel). 2023 Nov 14;16(22):7160. doi: 10.3390/ma16227160 (PMC10672246; doi:10.3390/ma16227160)
Supplement: Supplementary file 1 [file materials-16-07160-s001.zip › materials-2652873-supplementary.pdf]

Table S1. Algebraic expressions of functions $f(\alpha)$ proposed in the literature and their corresponding mechanisms [1–4].

No.	Symbol	$f(\alpha)$	Rate-determining mechanism
1. Chemical process or mechanism non-invoking equations			
1	F_2	$(1-\alpha)^2$	Chemical reaction
2	F_3	$(1-\alpha)^3$	Chemical reaction
3	F_n	$(1-\alpha)^n$	Chemical reaction
4	G_1	$(1/2)(1-\alpha)$	Chemical reaction
5	G_2	$(1/3)(1-\alpha)^2$	Chemical reaction
6	G_3	$(1/4)(1-\alpha)^3$	Chemical reaction
2. Acceleratory rate equations			
7	$P_{1/2}$	$2\alpha^{1/2}$	Nucleation
8	$P_{1/3}$	$3\alpha^{2/3}$	Nucleation
9	$P_{1/4}$	$4\alpha^{3/4}$	Nucleation
3. Sigmoidal rate equations or Avrami–Erofeev nuclei growth equations			
10	A_1	$(1-\alpha)$	Assumed random nucleation and its subsequent growth
11	$A_{3/2}$	$(3/2)(1-\alpha)[-\ln(1-\alpha)]^{1/3}$	Assumed random nucleation and its subsequent growth
12	A_2	$2(1-\alpha)[-\ln(1-\alpha)]^{1/2}$	Assumed random nucleation and its subsequent growth
13	A_3	$3(1-\alpha)[-\ln(1-\alpha)]^{2/3}$	Assumed random nucleation and its subsequent growth
14	G_4	$(1/2)(1-\alpha)[-\ln(1-\alpha)]^{-1}$	Assumed random nucleation and its subsequent growth, n=2
15	G_5	$(1/3)(1-\alpha)[-\ln(1-\alpha)]^{-2}$	Assumed random nucleation and its subsequent growth, n=3
16	G_6	$(1/4)(1-\alpha)[-\ln(1-\alpha)]^{-3}$	Assumed random nucleation and its subsequent growth, n=4

4. Deceleratory rate equations			
4.1. Phase boundary reaction			
17	R_2	$2(1-\alpha)^{1/2}$	Contracting cylinder
18	R_3	$3(1-\alpha)^{2/3}$	Contracting sphere
4.2. Based on the diffusion mechanism			
19	D_1	$1/(2\alpha)$	One-dimensional diffusion
20	D_2	$[-\ln(1-\alpha)]^{-1}$	Two-dimension diffusion, Valensi diffusion model
21	D_3	$(3/2)(1-\alpha)^{2/3}\left[1-(1-\alpha)^{1/3}\right]^{-1}$	Three-dimensional diffusion, Jander diffusion model
22	D_4	$(3/2)\left[(1-\alpha)^{-1/3}-1\right]^{-1}$	Three-dimensional diffusion, Brounstein–Ginstling model
23	D_5	$(3/2)(1-\alpha)^{4/3}\left[(1-\alpha)^{-1/3}-1\right]^{-1}$	Three-dimensional diffusion, Zhuralev–Lesokin–Tempelman model

Reference

[1] Gao, R.; Liu, B.; Zhan, L.; Guo, J.; Zhang, J.; Xu, Z. Catalytic effect and mechanism of coexisting copper on conversion of organics during pyrolysis of waste printed circuit boards. J. Hazard. Mater. 2021, 403, 123465. https://doi.org/10.1016/j.jhazmat.2020.123465.

[2] Jiang, H.; Wang, J.; Wu, S.; Wang, B.; Wang, Z. Pyrolysis kinetics of phenol–formaldehyde resin by non-isothermal thermo-gravimetry. Carbon 2010, 48, 352–358. https://doi.org/10.1016/j.carbon.2009.09.036.

[3] Taghizadeh, M.T.; Yeganeh, N.; Rezaei, M. Kinetic analysis of the complex process of poly(vinyl alcohol) pyrolysis using a new coupled peak deconvolution method. J. Therm. Anal. Calorim. 2014, 118, 1733–1746. https://doi.org/10.1007/s10973-014-4036-4.

[4] Li, L.; Guan, C.; Zhang, A.; Chen, D.; Qing, Z. Thermal stabilities and the thermal degradation kinetics of polyimides. Polym. Degrad. Stab. 2004, 84, 369–373. https://doi.org/10.1016/j.polymdegradstab.2003.11.007.
